# Publication audit & ever increasing problems faced by editors of Biomedical Journals

**DOI:** 10.12669/pjms.38.7.6920

**Published:** 2022

**Authors:** Shaukat Ali Jawaid

Problems and difficulties faced by Editors of Biomedical Journals in Pakistan continue to increase with every passing day. Devaluation of the Pakistani currency has resulted in tremendous increase in cost of production. Poor quality of manuscripts received for publication from within the country, lack of efficient reviewers, pressure by authors who are known as the most dangerous pressure group which the editors have to face as they wish to get their papers published soon after submission are some others difficulties which are mounting and it has become almost impossible for the editors to please everyone.^1^,^2^ Failure to withstand these pressures is one of the most important reasons why most of the journals published by medical institutions and professional specialty organizations have failed to improve the quality of the manuscripts they accept for publication and maintain the standard of their journal and get International recognition and indexation in important databases.

We keep on publishing guidelines for the authors, follow an author friendly policy and sometime ago highlighted and documented the reasons for not accepting the papers for further processing and how to get published in a standard peer review medical journal.^3^.^4^ An effort is made to ensure transparency at every level but still it fails to satisfy some of the authors who resort to levelling various allegations, send e mails which one would like to avoid reading let alone responding to them. Our experience shows that most of the authors who are most impatient and want quick publication seldom bother to carefully read and follow instructions for authors on the journal website. In many cases, even those manuscripts accepted for processing suffer from lot of deficiencies which are pointed out during Editor’s Triage and initial screening. The authors are advised to rectify them and resubmit but sometime they get the impression as if their manuscript has been approved for publication which is not true. In fact, since they are not fully familiar with the whole publication process, they get a wrong impression of acceptance. They start sending e mails asking for the publication date but when they are told that it will now go for external review, they get disappointed.

In order to help the authors and accelerate the peer review process, we now ask the authors to suggest two to three potential reviewers for their study. However, we make it clear that we might use any one of them along with some reviewers from our reviewer’s database and then look at their comments, edit them and then communicate it to the authors to revise their manuscripts. The suggested reviewers are also communicated guidelines on how to review a manuscript. It is also made absolutely clear that they should suggest only those as reviewers who are keen, interested, have worked in those particular areas and have the desired expertize to review the manuscript. Furthermore, their comments will be reviewed by the Editorial Team which may or may not accept their recommendations as they will be added to the comments received from other reviewers. The authors are conveyed all the comments in a combined e mail and they are asked to make the necessary changes in the file which is sent to them with instructions to make changes and highlight them. They are further instructed to ensure that the revised manuscript is accompanied with a covering note giving details of how they have responded to the reviewer’s comments and suggestions. Those manuscripts which require extensive revision are again sent to the same reviewers for their further input. However, in some cases what happens is that the authors even do not bother to read the e mail carefully, do not follow the instructions, send a new file with the changes, additions. It is not possible to format the whole manuscript once again as it takes a couple of hours and all this results in further delay in the processing of the manuscripts.

Most often reviewers have to be reminded to expedite the review. The authors must understand that the Editorial Team can edit and correct their manuscripts but they should not be expected to rewrite the manuscripts. If the English language is extremely poor and presentation is also disappointing, such papers certainly cannot be accepted. In order to avoid any inconvenience, we even ask the authors to send us the structured abstract of their paper by e mail and get a feedback within few days whether we can process their paper or not. They won’t have to lose the processing fee which is non-refundable once the paper has been submitted on the journal website with all the documents. The authors are supposed to provide structured abstract with all the authors, their affiliation and individual contribution in the study but most often they do not provide all this information. Provision of this detailed information along with structured abstract is essential for taking a decision as we are very careful about the menace of gift authorship that is why we most often do not allow more than four authors in single center studies. If the authors are told that the topic of their study is interesting, it does not mean it will also be accepted for publication because it all depends on the quality of presentation as well.

Further more it is not uncommon to receive requests for fast track processing of the manuscripts by the authors particularly those who are enrolled as PhD students in various universities despite the fact that it has been made clear on our website that currently facility of fast track processing is not available. Authors are asked to plan for at least six to eight months from the date of submission and those who are in a hurry, should submit their manuscripts to some other journal. The request is for immediate publication as they have got two to three months left. They already knew from the very first day that they need a publication based on their Thesis but they do not take this seriously, waste precious time, at times it is the lack of guidance by their unwilling supervisors as well. But editors cannot compensate for all the time lost. These PhD scholars need to plan it and ensure completion of their research project in time and submit in time for publication. Sometimes they insist that just give us the acceptance letter and the paper may be published when it is feasible but little do they realize that an acceptance letter can only be issued when the whole review process is completed and it takes couple of months. Every editor is also keen to quickly publish good quality studies which can get more citations, hence the researchers need to put emphasis on quality of their studies and innovation rather than undertaking routine work.

In order to know our strength and weaknesses, we always do our annual publication audit to find out the countries, regions from where we attract authors, the number of manuscripts being received for publication.^5^,^6^ An in depth review does reflect the trend. During the Year under review i.e. 2021, we received one thousand nine hundred thirty-four manuscripts. After initial screening, one thousand three hundred thirty-three were not accepted for further processing for various reasons. Nine manuscripts were rejected because of plagiarism, ten were withdrawn by the authors as we could not publish it within the time period they were expecting or had asked for. The number of papers published during the year were three hundred eighty three. Table-I.

**Table-I:** PJMS manuscripts statistics of 2021 at a Glance.

Total Published Articles:	383
Articles not accepted for further processing	1333
Total Articles Rejected due to Plagiarism	9
Articles Withdraw by Authors	10
Under Process:	199
Total Received Articles:	1934

A critical analysis further revealed that largest number of manuscripts received from overseas four hundred and two were from China followed by two hundred sixty-five from Turkey, one hundred three from Saudi Arabia. Table-II. Nine hundred thirty-five papers were received from Pakistan. As expected the largest number of papers from within the country were from the major cities, i.e. Karachi three hundred twenty-two, Lahore one hundred ninety-seven, Islamabad one hundred eighteen, Multan sixty-four, Peshawar sixty-two, Rawalpindi forty-nine, and Faisalabad twenty-two. Table-III. The number of papers being published after peer review from China has steadily increased from sixty-six in the Year 2000 to one hundred seventy-six in 2021. However, the number of papers published from Pakistan decreased from two hundred forty-one in 2000 to one hundred fifty-five in the Year 2021. Publications from Saudi Arabia also decreased from thirty-five in 2000 to twenty-two in the year 2021 because of the problems the authors have to face while transferring publication charges. Table-IV.

**Table-II:** Country wise submissions during 2021.

Country	Total
Afghanistan	1
Algeria	3
Argentina	1
Australia	1
Azerbaijan	1
Bangladesh	1
China	402
Christmas Island	1
Cyprus	6
Egypt	9
Ethiopia	4
France	1
India	16
Indonesia	19
Iran	49
Iraq	29
Ireland	5
Italy	1
Japan	1
Jordan	6
Kazakhstan	1
Korea	8
Malaysia	10
Nigeria	2
Norway	3
Oman	1
Pakistan	935
Qatar	1
Romania	2
Russia	10
Saudi Arabia	103
Senegal	1
Seychelles	2
Slovenia	1
South Africa	4
Spain	1
Sri Lanka	3
Sudan	5
Thailand	2
Turkey	265
Turks and Caicos Islands	1
Ukraine	1
United Arab Emirates	3
United Kingdom	6
United States of America	3
Uzbekistan	1
Vietnam	2

**Grand Total**	**1934**

**Table-III:** City wise submissions from Pakistan during 2021.

City	Total
Abbottabad	2
Azad Kashmir	2
Bahawalnagar	1
Bahawalpur	11
Chakdara	1
Chakwal	1
D.G. Khan	1
D.I. Khan	1
Faisalabad	22
Gilgit	3
Gujranwala	4
Gujrat	5
Gwadar	3
Hazara	1
Hyderabad	6
Islamabad	118
Jamshoro	12
Karachi	322
Kharian Cantt	1
Khuzdar	1
Kohat	1
Lahore	197
Larkana	2
Liaqatpur	1
Mansehra	4
Mardan	4
Multan	64
Muzaffarabad	1
Nawabshah	1
Nowshera	2
Okara	1
Peshawar	62
Quetta	6
R.Y. Khan	1
Rabwah	2
Rawalakot	1
Rawalpindi	49
Sahiwal	8
Sargodha	5
Sialkot	1
Sukkur	1
Swat	2
Turbat	1

**Total**	**935**

**Table-IV:** Manuscript published by Pak J Med Sci (2011 – 2021).

Country	2011	2012	2013	2014	2015	2016	2017	2018	2019	2020	2021
Algeria						1			1		
Australia						1	1	1	1		
Bahrain			1	1							
Bangladesh	4	4		1	1	1		1			
Canada										1	
Cameroon											
China	18	34	80	89	65	66	54	43	46	66	176
Cyprus		1	1					3	1		1
Fiji					1						
Germany			1								
India	1		1		1	1		1	1		
Iran	78	63	70	14	12	11	10	3	8	4	
Iraq	3	2	3	1		1		2	3	1	1
Ireland										1	
Japan							1				1
Jordan		1							1		1
Kenya		1									
Korea	2	1	3	2	5	7	2	2	1		1
Kuwait		1									
Malaysia	9	3	9	7	10	6	1	1		2	2
Nigeria	9			4	4	1				1	
Norway										1	4
Oman		1			1					1	
**Pakistan**	**93**	**65**	**91**	**93**	**106**	**135**	**163**	**150**	**168**	**241**	**155**
Qatar											1
Palestine			3	1	2						
Philippines						1					
Poland				2	4						
Romania			1	1	3	1	1	1		1	
Saudi Arabia	6	16	17	19	32	23	21	24	32	35	22
Serbia							1	1	1	1	
South Africa	2	1	6	2		1				1	
Sudan											
Taiwan	2	2	3								
Thailand								2			
Turkey	74	37	38	60	65	63	49	72	60	28	12
UAE	1	1			2		1	3		1	1
Uganda		1									
UK	5	1	2		1	1	1	1	1	2	3
USA				4			2			1	2

Total	307	236	330	301	315	321	308	311	325	389	383

**Table-V:** Category Wise Manuscript Published in 2021.

Category	Jan-Feb 2021	Mar-Apr 2021	May-Jun 2021	Jul-Aug 2021	Sep-Oct 2021	Wuhan Issue 2021	Nov-Dec 2021	Total
Case Report		3	1	2	1		2	9
Commentary				1			1	2
Correspondence	1	1	1	2	1		2	8
Editorial			1	1		1	1	4
Guest Editorial	1				5	1		7
Leading Article			1				1	2
Letter	1							1
Original Article	53	57	53	50	47	32	51	343
Review Article			1	2			1	4
Short Communication					1		1	2
Special Communications					1			1

Total	56	61	58	58	56	34	60	383

**Table-VI:** Country Wise Manuscript Published in 2021.

Country	Jan-Feb 2021	Mar-Apr 2021	May-Jun 2021	Jul-Aug 2021	Sep-Oct 2021	Wuhan Issue 2021	Nov-Dec 2021	Total
China	43	32	22	14	15	32	18	176
Cyprus							1	1
Iraq				1				1
Japan				1				1
Jordan		1						1
Korea				1				1
Malaysia			1	1				2
Norway	1		1	1	1			4
Pakistan	12	24	29	27	32	2	29	155
Qatar							1	1
Saudi Arabia		3	4	5	2		8	22
Turkey				5	5		2	12
United Arab Emirates							1	1
United Kingdom		1	1		1			3
United States of America				2				2

Total	56	61	58	58	56	34	60	383

Original articles were the largest number of papers, three hundred forty-three published during the Year 2021 followed by case reports nine, correspondence eight, Guest Editorials seven while only four review articles were accommodated during the period under review. During the year 2021 we also published a special issue to show case the research being conducted at Wuhan Institute of Technology in China.^7^

In order to improve the quality of research and manuscripts from Pakistan, it is essential that every medical institution in Pakistan keeps on organizing regular workshops on scientific writing. Keeping in view the number of undergraduate medical students and postgraduates in affiliated teaching hospitals who all need to publish, the Medical Education Department in every medical institution should organize at least two workshops every month, one for undergraduate and one for postgraduates. If they do not have trained professionals within the faculty, they can ask for help and assistance from Pakistan Association of Medical Editors (PAME), Editors of various biomedical journals which are available in almost every big city. They can be given some honorary affiliation if it works. Dept. of Medical Education can also help the authors if the human resource is available to review, help improve the manuscripts before they are submitted to medical journals for publication which will also help to minimize trauma to these manuscripts. However, it also depends on the interest of the head of the institution. If they are interested, they can work out some plans, otherwise the problems of the researchers, budding authors won’t be solved and the editor’s worries will also multiply.
